# A randomised phase 2b study comparing the efficacy and safety of belotecan vs. topotecan as monotherapy for sensitive-relapsed small-cell lung cancer

**DOI:** 10.1038/s41416-020-01055-5

**Published:** 2020-11-16

**Authors:** Jin-Hyoung Kang, Ki-Hyeong Lee, Dong-Wan Kim, Sang-We Kim, Hye Ryun Kim, Joo-Hang Kim, Jin-Hyuk Choi, Ho Jung An, Jin-Soo Kim, Joung-Soon Jang, Bong-Seog Kim, Heung Tae Kim

**Affiliations:** 1 The Catholic University of Korea Seoul St. Mary’s Hospital, Seoul, South Korea; 2 Chungbuk National University Hospital, Cheongju, South Korea; 3 Seoul National University Hospital, Seoul, South Korea; 4 Asan Medical Center, University of Ulsan College of Medicine, Seoul, South Korea; 5 Yonsei Cancer Center, Division of Medical Oncology, Yonsei University College of Medicine, Seoul, South Korea; 6 CHA University Bundang Medical Center, Seongnam, South Korea; 7 Ajou University Hospital, Suwon, South Korea; 8 The Catholic University of Korea St. Vincent’s Hospital, Seoul, South Korea; 9 Seoul National University Boramae Medical Center, Seoul, South Korea; 10 Chung-Ang University College of Medicine, Seoul, South Korea; 11 Veterans Health Service Medical Center, Seoul, South Korea; 12 National Cancer Center, Goyang, South Korea

**Keywords:** Small-cell lung cancer, Lung cancer

## Abstract

**Background:**

This study compared the efficacy/safety of the camptothecin analogues belotecan and topotecan for sensitive-relapsed small-cell lung cancer (SCLC).

**Methods:**

One-hundred-and-sixty-four patients were randomised (1:1) to receive five consecutive daily intravenous infusions of topotecan (1.5 mg/m^2^) or belotecan (0.5 mg/m^2^), every 3 weeks, for six cycles. Main outcomes were objective response rate (ORR), disease control rate (DCR), progression-free survival (PFS), overall survival (OS), tolerability and toxicity. The study statistical plan was non-inferiority design with ORR as the endpoint.

**Results:**

In the belotecan vs. topotecan groups, ORR (primary endpoint) was 33% vs. 21% (*p* = 0.09) and DCR was 85% vs. 70% (*p* = 0.030). PFS was not different between groups. Median OS was significantly longer with belotecan than with topotecan (13.2 vs. 8.2 months, HR = 0.69, 95% CI: 0.48–0.99), particularly in patients aged <65 years, with more advanced disease (i.e., extensive-stage disease, time to relapse: 3–6 months), or Eastern Cooperative Oncology Group performance status 1 or 2. More belotecan recipients completed all treatment cycles (53% vs. 35%; *p* = 0.022).

**Conclusions:**

The efficacy/safety of belotecan warrants further evaluation in Phase 3 trials. Belotecan potentially offers an alternative to topotecan for sensitive-relapsed SCLC, particularly in patients aged <65 years, with more advanced disease, or poor performance.

## Background

Small-cell lung cancer (SCLC) is a highly aggressive carcinoma with early metastasis and poor prognosis, characterised by low differentiation, high mutational burden and high count of circulating tumour cells.^1^ Although most SCLC patients initially respond to first-line platinum-based chemotherapies, 80% of patients with limited-stage disease (LD) relapse within a year post treatment, as do nearly all patients with extensive-stage disease (ED).^2^ Treatment options for SCLC are very limited because the tumours become increasingly resistant to first-line chemotherapy. Topotecan, a topoisomerase I inhibitor, is currently the only single-agent drug approved by the US Food and Drug Administration (FDA) as a second-line treatment for relapsed SCLC. Its efficacy is more reliable in patients with sensitive-relapse (time to relapse ≥3 months after completion of first-line chemotherapy) than those with resistant-relapse (time to relapse <3 months).^3^

Belotecan is a new camptothecin analogue topoisomerase I inhibitor. In animal models, belotecan has demonstrated greater anti-tumour efficacy and wider therapeutic margins than topotecan.^4^ In multiple Phase 2 clinical trials, belotecan monotherapy demonstrated initial efficacy and favourable safety profiles for the treatment of relapsed SCLC.^5–7^ However, there has been no head-to-head comparison between belotecan and topotecan. Therefore, the primary objective of this randomised clinical trial was to compare the efficacy and safety of topotecan vs. belotecan as monotherapy for relapsed SCLC. The secondary objectives were to provide further information on the use of both drugs (i.e., overall survival [OS] in different subgroups of patients), and to identify prognostic factors for OS and progression-free survival (PFS) in patients with sensitive-relapsed SCLC.

## Patients and methods

### Eligibility

Patients were 18 years or older, with histologically or cytologically confirmed SCLC at either LD or ED, who had developed sensitive-relapse (time to relapse ≥3 months after completion of first-line chemotherapy). Patients with time to relapse ≥6 months who chose clinical trial treatments, other than rechallenge with their original chemotherapies, were included. Additional eligibility criteria included at least one unidimensionally measurable lesion according to the Response Evaluation Criteria in Solid Tumours version 1.1 (RECIST 1.1), Eastern Cooperative Oncology Group performance status (ECOG PS) of 0–2, expected survival of 3 months or longer (based on the treating physician’s judgment), and normal haematology (haemoglobin [Hb] ≥9.0 g/dL) and biochemistry test results (or abnormal test results which were clinically insignificant based on the treating physician’s reasonable medical judgment). Patients with symptomatic brain metastasis within 3 months prior to study entry were excluded.

### Study design

This was a Phase 2b, multicentre, randomised, open-label, parallel-group study. Randomisation was conducted using the restricted block randomisation method. The primary endpoint was objective response rate (ORR), which is the percentage of patients with best overall response (OR) of complete response (CR) and partial response (PR). Secondary endpoints were (1) disease control rate (DCR), defined as the percentage of patients with best OR of CR, PR, and stable disease (SD); (2) PFS, defined as the time elapsed between randomisation and tumour progression or death from any cause; and (3) OS, defined as the time from randomisation to death from any cause.

### Treatment plan

Topotecan (Hycamtin, GlaxoSmithKline, London, UK) or belotecan (Camtobell, CKD-602, Chong Kun Dang Pharmaceutical Corp., Seoul, South Korea) were administered as monotherapy. A 21-day cycle of treatment consisted of five consecutive daily intravenous infusions of topotecan (1.5 mg/m^2^) or belotecan (0.5 mg/m^2^), infused over 30 min. All patients were intended to receive six cycles of treatment.

### Treatment modification

Treatment was suspended (maximum 2 weeks) in any of the following circumstances: (1) absolute neutrophil count (ANC) <1500 per μL, (2) platelet count <100,000 per μL, (3) febrile neutropenia, (4) non-haematological grade 3/4 adverse event (AE), or (5) topotecan-related renal toxicity (creatinine clearance <40 mL/min). Re-evaluation was conducted within 2 weeks of treatment suspension.

The daily dose was reduced by 0.1 mg/m^2^ for belotecan and 0.25 mg/m^2^ for topotecan, if re-evaluation results showed any of the following: (1) ANC between 1000–1500 per μL, (2) platelet count between 75,000 and 100,000 per μL, (3) febrile neutropenia amelioration, or (4) non-haematological grade 3/4 AE decreased to grade 1/2. The daily dose of topotecan was reduced to 0.75 mg/m^2^ if creatinine clearance was between 20 and 40 mL/min. Treatment was discontinued in the following circumstances: (1) ANC < 1000 per μL, (2) platelet <75,000 per μL, (3) non-amelioration of febrile neutropenia, (4) persistent grade 3/4 non-haematological AE, or (5) creatinine clearance <20 mL/min.

Febrile neutropenia was defined as ANC < 1000 per μL and body temperature ≥38.5 °C. Non-haematological grade 3/4 AEs did not include alopecia, anorexia, nausea, or vomiting.

### Tumour response evaluation

Tumour size was calculated as the sum of the longest diameter of the target lesions using the same imaging system (e.g., CT, MRI, and chest X-ray) utilised at screening and post-randomisation. OR and best OR were evaluated according to the RECIST 1.1 guidelines by an independent blinded radiologist. OR was evaluated every two cycles unless the patient previously showed CR or PR, in which case OR was evaluated no earlier than 4 weeks following the event.

### Tolerability and toxicity evaluation

Tolerability was measured by the relative dose intensity (RDI) of each cycle, with RDI defined as the amount of a particular chemotherapy given over a specific time in relation to what was ordered, and calculated using the following equation:

$${\mathrm{RDI}} = \left( {\frac{{{\mathrm{Actual}}\,{\mathrm{total}}\,{\mathrm{dose}}}}{{{\mathrm{Actual}}\,{\mathrm{total}}\,{\mathrm{injection}}\,{\mathrm{days}}}}\bigg/\frac{{{\mathrm{Planned}}\,{\mathrm{total}}\,{\mathrm{dose}}}}{{{\mathrm{Planned}}\,{\mathrm{total}}\,{\mathrm{injection}}\,{\mathrm{days}}}}} \right) \times 100\% .$$

For each cycle, toxicity was evaluated through physical examination and ECOG PS on day 1, and haematology and biochemistry tests were evaluated on day 21. AEs were graded based on the Common Terminology Criteria for Adverse Events version 4.0 (CTCAE 4.0). In accordance with FDA guidelines, serious AEs were defined as events where the patient’s outcome was death, life-threatening, hospitalisation (initial or prolonged), or disability or permanent damage.^8^

### Discontinuation of clinical trial treatment

Clinical trial treatment was terminated in the following circumstances: (1) symptomatic deterioration before completion of all six cycles of treatment, (2) more than two instances of treatment suspension or dose reduction, (3) patient’s voluntary withdrawal of consent, (4) protocol violation in enrolment, randomisation, or study compliance, (5) change to other treatments, (6) treating physician’s evaluation that the risks of AEs outweighed the benefits of treatment.

### Post-trial treatment

Post-clinical trial treatment and care was deferred to the discretion of the treating physician. If post-trial treatment was topotecan or belotecan, monitoring and recording of adverse drug reactions and serious AEs continued.

### Statistical considerations

Sample size (*N*) was determined as the number of patients needed to achieve a power of 80% (1−*β* = 0.80) to conclude non-inferiority of belotecan to topotecan, at a one-sided type I error of 5% (*α* = 0.05). The equation used to calculate sample size was adapted from a previous publication^9^ as follows:

$$N \ge 2 \times \left( {\frac{{Z_\alpha + Z_{1 - \beta }}}{\delta }} \right)^2 \times p \times (1 - p) \times \frac{1}{{1 - d,}}$$

where Z is the normal distribution function, δ is the non-inferiority margin which was determined to be 19% based on the range of published ORR (0–37%) of topotecan^3^ and clinical considerations, p is the estimated ORR of topotecan set at 25%,^3^ and *d* is the estimated drop-out rate set at 20%. The main statistical assumption of this study was that the ORR in the experimental arm would be non-inferior to that in the control arm under the selected non-inferiority margin (−0.19, or 19%).

Safety analysis included all patients who received at least one dose of treatment (safety data set). Efficacy analysis included all patients who underwent at least one tumour response evaluation (efficacy data set). Kaplan–Meier survival analysis and the log-rank test were used to compare PFS and OS between treatment groups. Stepwise Cox regression analysis was used to identify significant prognostic factors for PFS and OS among the following categorical variables: treatment, age, sex, disease stage at diagnosis, disease stage at enrolment, ECOG PS, time to relapse, RDI, and baseline Hb. Stratified Cox regression was used to compare topotecan and belotecan for PFS and OS in subgroup populations stratified by sex, age, time to relapse, baseline Hb, ECOG PS, disease stage, RDI, metastatic status and prior radiotherapy history.

## Results

### Patients

Between September 2010 and December 2017, 164 patients from 14 hospitals were randomly assigned (1:1) to receive either topotecan or belotecan (Supplementary Fig. S1). Three patients withdrew consent before treatment initiation leaving 161 intention-to-treat patients (topotecan: *n* = 81; belotecan: *n* = 80) for the safety analysis. Thirteen patients dropped out before their first tumour response evaluation, leaving 148 patients (topotecan: *n* = 76; belotecan: *n* = 72) for the efficacy analysis. Baseline characteristics of patients were similar between groups (Supplementary Table S1).

### Treatment exposure

Treatment exposure in patients from the safety data set is presented in Table 1. Compared to the topotecan group, patients in the belotecan group received more treatment cycles (belotecan vs. topotecan: 4.4 vs. 3.7 cycles; independent *t*-test, *p* = 0.021), but fewer patients dropped out in this group, particularly after the first efficacy evaluation (belotecan vs. topotecan: 8% vs. 22%; Chi-square test, *p* = 0.029). Compared to the topotecan group, more patients in the belotecan group completed more than two cycles of treatment, (belotecan vs. topotecan: 75% vs. 60%; Chi-square test, *p* = 0.049). In particular, more patients in the belotecan group finished all six cycles than in the topotecan group (belotecan vs. topotecan: 53% vs. 35%; Chi-square test, *p* = 0.022). For all patients who completed the first two cycles, the RDIs of the first two treatment cycles were significantly higher in the belotecan group (88–90%) than in the topotecan group (81–85%, independent *t*-test, *p* < 0.05). However, for patients who finished more than two treatment cycles, the RDIs in the last four cycles were not different between the two groups. The RDIs per cycle were marginally higher in the belotecan group (85%) than in the topotecan group (81%) in patients aged <65 years (independent t-test, *p* = 0.07) but were similar in patients aged ≥65 years (independent *t*-test, *p* = 0.60).

**Table 1:** Treatment exposure of patients in the safety data set.

	Topotecan (*n* = 81)	Belotecan (*n* = 80)	*p* value
Cycles received, mean ± sd	3.7 ± 2.0	4.4 ± 1.9	0.021^a^
Treatment completion, *n* (%)			
Yes	58 (72%)	66 (82%)	0.10^b^
No (drop-outs)	23 (28%)	14 (18%)	
Drop-out time, *n* (%)			
Before 1st efficacy evaluation	5 (6%)	8 (10%)	0.029^b^
After 1st efficacy evaluation	18 (22%)	6 (8%)	
Patients received 1–6 cycles, *n* (%)			
1 cycle	13 (16%)	9 (11%)	0.027^b^
2 cycles	19 (23%)	11 (14%)	
3 cycles	7 (9%)	2 (3%)	
4 cycles	9 (11%)	15 (19%)	
5 cycles	5 (6%)	1 (1%)	
6 cycles	28 (35%)	42 (53%)	
RDI^c^ by cycle, (mean ± sd)%			
Cycle 1	(85 ± 14)%	(90 ± 31)%	0.004^a^
Cycle 2	(81 ± 19)%	(88 ± 14)%	0.018^a^
Cycle 3	(80 ± 17)%	(80 ± 16)%	0.94^a^
Cycle 4	(81 ± 15)%	(82 ± 15)%	0.52^a^
Cycle 5	(80 ± 17)%	(82 ± 19)%	0.25^a^
Cycle 6	(91 ± 13)%	(93 ± 14)%	0.48^a^
Average RDI^c^ per cycle, (mean ± sd)%			
<65 years	(81 ± 12)%	(85 ± 9)%	0.07^a^
≥65 years	(83 ± 13)%	(81 ± 13)%	0.60^a^

*RDI* relative dose intensity.

^a^*p* values were obtained using independent two sample *t*-test.

^b^*p* values were obtained using Chi-square test, *p* < 0.05 indicates that the distribution of treatment cycles differed between groups.

^c^RDI = [(Actual total dose/actual total injection days)/(Planned total dose/planned total injection days)] × 100%.

### Post-trial treatment

Three patients in each group received additional clinical trial treatments after the scheduled treatment was completed. In the topotecan group, one patient received one additional cycle, one received two additional cycles, and one received four additional cycles of treatment. In the belotecan group, two patients received one additional cycle, and one received three additional cycles. After the completion of clinical trial treatments, about half of the patients (topotecan, *n* = 35; belotecan, *n* = 47) received other chemotherapies (e.g., etoposide/platinum, irinotecan/platinum) during the follow-up period, and two patients in the belotecan group received immunotherapy (pembrolizumab) (Supplementary Table S2).

### Efficacy

**Fig. 1:**
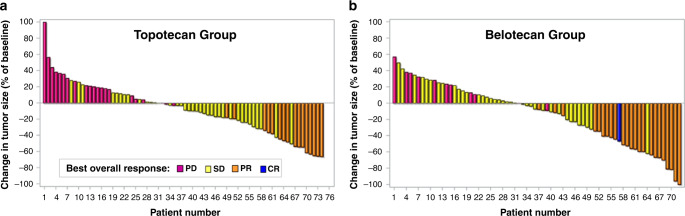
Waterfall plots demonstrating the efficacy of belotecan and topotecan monotherapy in patients with sensitive-relapsed SCLC. Maximum percentage changes in tumour size (sum of the longest diameters of target-lesions) from baseline, in topotecan (**a**) and belotecan (**b**) groups. Colours indicate best overall responses determined from the start of treatment until disease progression/recurrence. *p* values were obtained from Chi-square tests for comparisons of ORR and DCR between groups.

**Fig. 2:**
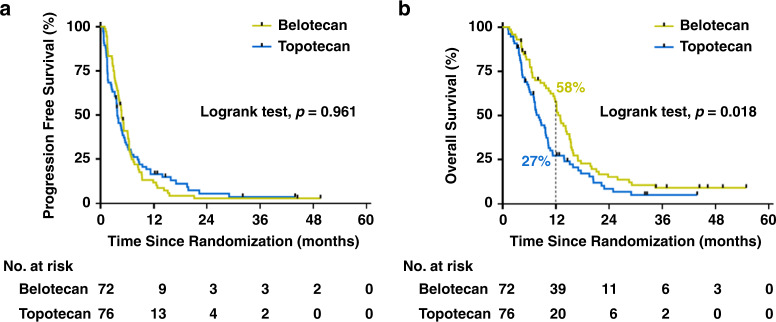
Survival curves for belotecan and topotecan. Kaplan–Meier curves showing **a** PFS and **b** OS for belotecan and topotecan groups. *p* values were obtained from log-rank tests for comparisons of PFS and OS between groups. Vertical dashed line indicates 1-year OS rate. ORR objective response rate, DCR disease control rate, PFS progression-free disease, OS overall survival.

Changes in target-lesion size from baseline and best OR are presented in Fig. 1a, b. During treatment, more patients in the belotecan group demonstrated ORs (PR+CR) compared to the topotecan group (belotecan vs. topotecan: *n* = 24 vs. *n* = 16). Non-inferiority was demonstrated for the between-group difference in ORR (primary endpoint; belotecan vs. topotecan: 33% vs. 21%, 95% confidence interval [CI] −0.0195 to 0.2651), but the between-group difference did not reach statistical significance (Chi-square test*, p* = 0.09). Supplementary Table S3 presents best OR data for the full analysis set, intention-to-treat (ITT), and modified ITT populations. In the ITT analysis, the 95% CI for the between-group difference in ORR was −0.0330 to 0.2282; as the one-sided CI for the difference in response rate of the two groups was −0.0330, which is larger than −0.19 (the non-inferiority tolerance limit), the anti-cancer efficacy of belotecan is non-inferior to that of topotecan, even in the ITT group. The DCR was significantly higher in the belotecan group compared to the topotecan group (belotecan vs. topotecan: 85% vs. 70%; Chi-square test*, p* = 0.030). Before treatment completion, disease progression was twice as high in the topotecan group (*n* = 23) than the belotecan group (*n* = 11). However, no significant difference in PFS was observed between groups (Fig. 2a). The median PFS was 4.8 months for belotecan and 3.8 months for topotecan (log-rank test, *p* = 0.961; HR = 1.65, 95% CI: 1.17–2.33). However, OS was significantly superior in the belotecan group (Fig. 2b). The median OS was 13.2 months for belotecan and 8.2 months for topotecan (log-rank test, *p* = 0.018; HR = 0.69, 95% CI: 0.48–0.99). The 12-month OS was 58% for belotecan and 27% for topotecan (Chi-square test*, p* = 0.0001).

**Fig. 3:**
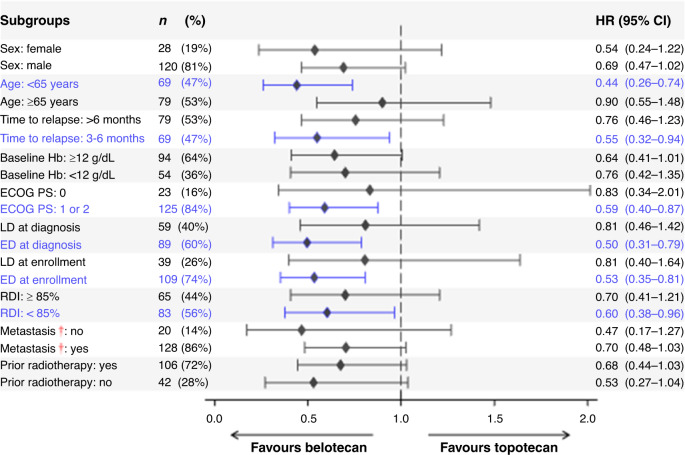
Forest plot showing HR of belotecan relative to topotecan for OS in different subgroups. HR (95% CI) < 1 indicates significantly longer survival in the belotecan group than in the topotecan group. Dagger indicates that patients with symptomatic brain metastasis within 3 months prior to study entry were excluded from this study. HR hazard ratio, ECOG PS Eastern Cooperative Oncology Group performance status, LD limited-stage disease, ED extensive-stage disease, RDI relative dose intensity.

Throughout the 7-year study, there were 126 documented deaths, and 22 patients with censored OS data (Fig. 2b), including seven patients who were lost to follow-up (belotecan vs. topotecan: *n* = 2 vs. *n* = 5) and 15 patients who were still alive (belotecan vs. topotecan: *n* = 9 vs. *n* = 6). The OS benefit of belotecan was particularly strong in the following patient subgroups: age <65 years, ED (either at diagnosis or enrolment), earlier relapse (time to relapse: 3–6 months), poorer performance status (ECOG PS 1 or 2), and RDI <85% (Fig. 3). Neither belotecan nor topotecan showed superior PFS in any subgroups (data not shown).

### Prognostic factors

Among the 148 patients included for prognostic factor analysis (efficacy data set; Table 2), the independent risk factors for PFS were earlier relapse (time to relapse: 3–6 months; Cox regression, HR = 1.65 and *p* = 0.004) and lower RDI (RDI < 85%; Cox regression, HR = 1.48 and *p* = 0.024). Belotecan was an independent protective factor (Cox regression, HR = 0.69 and *p* = 0.045) for OS. Earlier relapse, lower baseline Hb (<12 g/dL), and an ECOG PS of 2 were independent risk factors (Cox regression, HR = 2.07, 1.36, and 7.22, respectively, all *p* values < 0.05). Subgroup analysis showed that lower baseline Hb was associated with shorter OS only in patients with ED-SCLC (stratified Cox regression, *p* = 0.018), but not in patients with LD-SCLC.

**Table 2:** Stepwise Cox regression analyses for prognostic factors.

	Reference	HR (95% CI)	*p* value
Progression-free survival			
Time to relapse: 3–6 months	≥6 months	1.65 (1.17–2.33)	0.004
RDI: < 85%	≥85%	1.48 (1.05–2.09)	0.024
Overall survival			
Treatment type: belotecan	Topotecan	0.69 (0.48–0.99)	0.045
Time to relapse: 3–6 months	≥6 months	2.07 (1.42–3.03)	0.0002
ECOG PS: 1	0	1.36 (0.83–2.22)	0.22
ECOG PS: 2	0	7.22 (2.05–25.4)	0.002^a^
Baseline Hb: < 12 g/dL	≥12 g/dL	1.54 (1.05–2.26)	0.028
Baseline Hb: <12 g/dL_LD	≥12 g/dL_LD	1.87 (0.82–4.25)	0.13^b^
Baseline Hb: <12 g/dL_ED	≥12 g/dL_ED	1.64 (1.08–2.47)	0.018^b^

*ECOG PS* Eastern Cooperative Oncology Group performance status, *Hb* haemoglobin.

^a^Only three patients had an ECOG PS of 2.

^b^*p* values were calculated using stratified Cox regression analysis.

### Toxicity

The incidence of all grade AEs and grade 3/4 AEs did not differ between treatments (safety data set; Table 3). In both groups, the large majority of patients experienced grade 3/4 AEs, the most common being haematological disorders (≥10%), such as neutropenia, thrombocytopenia, and anaemia. Over 40% of patients in the belotecan group experienced serious AEs (*n* = 33) compared to ~50% of patients in the topotecan group (*n* = 43). Of note, 20% more patients with RDI < 85% in the topotecan group experienced serious AEs than their counterparts in the belotecan group (59% vs. 38%; Chi-square test*, p* = 0.048) (Table 3). One grade 5 AE (death from pneumonia) in the belotecan group was determined to be treatment related.

**Table 3:** Adverse events in patients in the safety data set.

	Grade 1–4		Grade 3/4	
	Topotecan (*n* = 81)	Belotecan (*n* = 80)	Topotecan (*n* = 81)	Belotecan (*n* = 80)
Summary, *n* (% of subgroup population)				
AEs				
RDI: ≥85%	37 (100%)	40 (100%)	30 (81%)	33 (83%)
RDI: <85%	43 (98%)	39 (98%)	40 (91%)	34 (85%)
Serious AEs^a^				
RDI: ≥85%	17 (46%)	18 (45%)	16 (43%)	15 (38%)
RDI: <85%	26 (59%)	15 (38%)	25 (57%)	14 (35%)
	*p* = 0.048^b^		*p* = 0.045^b^	
SOC^c^, *n* (% of group population)				
Investigations				
Neutropenia	67 (83%)	61 (76%)	59 (73%)	54 (68%)
Thrombocytopenia	43 (53%)	33 (41%)	36 (44%)	29 (36%)
Leukocytopenia	20 (25%)	18 (23%)	18 (22%)	16 (20%)
Blood and lymphatic system				
Anaemia	44 (54%)	37 (46%)	20 (25%)	22 (28%)
Febrile neutropenia^d^	1 (1%)	4 (5%)	1 (1%)	4 (5%)
Metabolism and nutrition				
Anorexia	36 (44%)	32 (40%)	3 (4%)	0 (0%)
Gastrointestinal system				
Nausea	30 (37%)	35 (44%)	1 (1%)	2 (2%)
Constipation	16 (20%)	18 (23%)	0 (0%)	0 (0%)
Vomiting	10 (12%)	14 (18%)	1 (1%)	1 (1%)
Diarrhoea	9 (11%)	12 (15%)	2 (3%)	1 (1%)
Abdominal pain	7 (9%)	10 (13%)	0 (0%)	0 (0%)
Mucositis oral	7 (9%)	10 (13%)	0 (0%)	0 (0%)
Respiratory, thoracic and mediastinal system				
Dyspnoea	20 (25%)	14 (18%)	3 (4%)	4 (5%)
Cough	9 (11%)	16 (20%)	0 (0%)	0 (0%)
Productive cough	9 (11%)	8 (10%)	1 (1%)	0 (0%)
General and administration site conditions				
Fatigue	20 (25%)	18 (23%)	3 (4%)	1 (1%)
Pain	10 (12%)	11 (14%)	0 (0%)	0 (0%)
Fever	8 (10%)	11 (14%)	0 (0%)	0 (0%)
Nervous system				
Dizziness	13 (16%)	14 (18%)	1 (1%)	1 (1%)
Headache	10 (12%)	8 (10%)	0 (0%)	0 (0%)
Skin and subcutaneous tissue				
Alopecia	12 (15%)	8 (10%)	0 (0%)	0 (0%)

*AE* adverse event, *RDI* relative dose intensity, *SOC* system organ class.

^a^Serious AEs: when the patient outcome is death, life-threatening, hospitalisation (initial or prolonged), or disability or permanent damage, in accordance with FDA guidelines.

^b^*p* value was obtained using Chi-square test, *p* < 0.05 indicates that in patients with RDI < 85%, belotecan resulted in significantly fewer serious AEs than topotecan.

^c^Only common (≥10%) AEs are presented.

^d^Febrile neutropenia: absolute neutrophil count <1000 per mm^3^ and fever ≥38.5 °C. Grade 3/4 febrile neutropenia-associated hospitalisation/prolonged hospitalisation occurred in 1 and 3 patients in the topotecan and belotecan groups, respectively.

## Discussion

This is the first randomised clinical trial to compare the efficacy and safety of belotecan vs. topotecan in SCLC patients. Dozens of randomised clinical trials have been conducted in an effort to discover safe and effective alternatives to the current standard second-line treatment, topotecan (Supplementary Table S4). However, no experimental drug has been reported to surpass topotecan for both efficacy and safety. Emerging and revolutionary cancer immunotherapies such as PD-L1 and PD-1 inhibitors have recently gained FDA approval as third-line treatments for metastatic SCLC. Combinations of PD-1 or CTLA-4 inhibitors with standard treatment have the potential to be first-line treatments for ED-SCLC,^10^ but the performance of immunotherapies as second-line or maintenance treatments has proven to be inadequate.^11,12^

Our study adopted the topotecan regimen (1.5 mg/m^2^, for 5 consecutive days, every 3 weeks) used by studies in a meta-analysis of 10 clinical trials, including more than a total of 800 patients with sensitive-relapsed SCLC.^3^ The topotecan results obtained in the current study closely mirror the findings of the meta-analysis; the ORRs were 21% vs. 21%, and 12-month OS rates were 27% vs. 27%, respectively. The meta-analysis and the current study had comparable incidences of grade 3/4 neutropenia (76% vs. 73%), thrombocytopenia (45% vs. 44%), and anaemia (29% vs. 25%). The topotecan group in our study served as quality control and its consistency with previous studies indicates the representativeness of our data to clinical settings for sensitive-relapsed SCLC.

For efficacy comparison, belotecan demonstrated only moderate (10–15%) improvements in ORR (primary endpoint) and DCR (secondary endpoint) compared to topotecan. Although non-inferiority was demonstrated for the between-group difference in ORR, the difference did not reach statistical significance (belotecan vs. topotecan: 33% vs. 21%; 95% CI, −0.0195 to 0.2651; Chi-square test*, p* = 0.09). PFS was not different between treatment groups. Strikingly, OS was 5 months longer in the belotecan group than in the topotecan group. The 1-year OS rate of the belotecan group was twice that of the topotecan group (58% vs. 27%). Considering that the numbers of patients who received the extended clinical trial treatments and the percentages of patients who received post-trial chemotherapies were not different between the two groups, and the OS difference remained relatively consistent over time, it is unlikely that the superior OS in the belotecan group was caused by factors outside the clinical trial treatment. Although more patients received post-trial chemotherapies in the belotecan group, this was because more patients survived in this group. Regarding the discrepancy between PFS and OS in this study, because PFS is a straightforward measure of therapy-related benefit, while OS is more of a reflection of tumour growth after treatment cessation (which is also regarded as the gold standard for cancer treatment efficacy),^13–15^ significantly improved PFS may not always lead to improved OS, or vice versa.^16^

Our subgroup analyses, while limited in their ability to draw firm conclusions, indicate that belotecan was superior to topotecan for OS in patients aged <65 years, patients with more advanced disease (i.e., ED, time to relapse: 3–6 months), and those with ECOG PS of 1 or 2 (Fig. 3). Elderly patients tend to benefit less from chemotherapy for SCLC because they tolerate it poorly.^17^ In this study, the RDI per cycle of older patients (≥65 years) was similar between treatment groups, but the RDI per cycle of younger patients (<65 years) was marginally higher in the belotecan group (Table 1), which likely accounts for belotecan’s superiority in the younger group. Belotecan’s longer OS in patients with more advanced disease may be partially due to its higher anti-tumour potency,^4^ particularly in tumours rich in *TP53* mutations.^18^ It is well-established that advanced SCLC has high levels of *TP53* mutation.^19,20^ Our subgroup OS analyses imply that the patient’s baseline characteristics may indicate whether belotecan would be preferable to topotecan on an individual basis.

We found several factors significantly associated with survival, including time to relapse, disease stage, and baseline Hb (Table 2). Time to relapse was the most important prognostic factor for both PFS and OS. Relapsed SCLC is usually classified into resistant and sensitive types using the cut-point of 3 months.^21,22^ Our data suggest that within the same category of sensitive-relapse, risk of disease progression or death is 1.5–2 fold higher for patients with earlier sensitive-relapse (time to relapse: 3–6 months) than patients with later sensitive-relapse (time to relapse: >6 months). Time to relapse should be the main consideration when selecting second-line treatment. According to National Comprehensive Cancer Network (NCCN) and Korean Society for the Study of Lung Cancer (KASLC) guidelines,^23^ topotecan is the first recommendation for patients with earlier sensitive-relapse. However, our results indicate that belotecan may be more beneficial than topotecan in this subpopulation. Patients with an ECOG PS of 2 showed a remarkably higher risk (HR = 7.22) of death compared to those with an ECOG PS of 0. It should be noted that, although this result is consistent with previous studies,^24^ the HR may not be an accurate estimation because the number of patients with an ECOG PS of 2 in this study is very small (*n* = 3). ED is another established prognostic factor for poor OS.^25–27^ However, in this study ED was not significantly associated with shorter OS. This is probably due to the two-fold greater OS benefit of belotecan over topotecan in patients with ED-SCLC. Anaemia is a risk factor for poor chemotherapy outcomes.^28^ Nadir Hb post-chemotherapy and survival in NSCLC patients without baseline anaemia are significantly associated.^29^ However, a large-scale retrospective study found no association between anaemia and OS for LD-SCLC.^30^ In the current study, the subgroup analysis indicates that anaemia is a significant prognostic factor for poorer OS only in patients with ED-SCLC.

A single-arm clinical trial of belotecan monotherapy in patients with sensitive-relapsed SCLC reported a lower ORR (22%) than our study (33%).^5^ The discrepancy may reflect the fact that treatment with irinotecan occurred prior to enrolment in all patients in the study by Jeong et al.,^5^ but only in 10% of patients in the current study. This may support Jeong’s concern about cross-resistance to belotecan following irinotecan treatment.^5^ It is known that tumours may develop acquired resistance to irinotecan via multiple mechanisms, including down-regulation of topoisomerase I expression or alteration of the topoisomerase I structure.^31^ Whether these mechanisms also drive belotecan resistance needs to be investigated. Jeong’s study reported median PFS and OS values (4.7 and 13.1 months, respectively), which are very similar to those in our study (4.8 and 13.2 months, respectively). This suggests that any acquired resistance post-irinotecan treatment is unlikely to significantly influence belotecan’s survival benefits.

Recently, combination treatments have demonstrated encouraging tumour responses and/or survival benefits for relapsed SCLC,^32,33^ but safety concerns, including significantly more grade 3/4 toxicities and serious AEs with combination treatments, have hampered their application. In the current study, despite the fact that patients in the belotecan group received on average nearly one more treatment cycle than those in the topotecan group, the two treatments showed similar incidences of AEs. In addition, the RDIs were significantly higher in the belotecan group than in the topotecan group only for the first two cycles, then they became similar. This may be because more patients with low RDIs dropped out of the topotecan group than the belotecan group during the first two cycles. It is unsurprising because those who required considerable dose reduction (RDI < 85%) in the topotecan group had a significantly higher risk of serious AEs than those in the belotecan group (Table 3). More consistent treatment resulting from the better tolerability of belotecan explains why patients with RDI < 85% survived significantly longer in that group.

There are several limitations to this study. First, it was only conducted in Korean patients. Asian and European ethnicities may differ in response to systemic treatments for SCLC.^34^ Whether there are ethnic differences for belotecan treatment needs further investigation. Second, this was an open-label study. However, to minimise potential bias caused by the open-label design, all tumour response evaluations were conducted by an independent central reviewer who was blinded to treatment-type. Third, patients’ blood and tumour tissue samples were not preserved which makes retrospective studies to investigate biomarkers of prognosis and resistance impossible.

In summary, based on the non-inferiority primary endpoint, ORR, belotecan demonstrated efficacy and safety compared with topotecan, which warrants further evaluation in Phase 3 trials for the treatment of relapsed SCLC. We propose that belotecan has the potential to be considered as an alternative second-line treatment to topotecan for sensitive-relapsed SCLC and may be recommended as the preferred treatment for patients under 65 years old, patients with more advanced disease (i.e., ED, time to relapse: 3–6 months), or those with poor performance status (ECOG PS: 1 or 2). We expect these results to contribute to the advancement of personalised therapy for relapsed SCLC.

## Supplementary information

Supplementary File

## Data Availability

The data are available for all study authors. The datasets used and analysed during the current study are available from the corresponding author on reasonable request.
