# Supplementary material for: A randomised phase 2b study comparing the efficacy and safety of belotecan vs. topotecan as monotherapy for sensitive-relapsed small-cell lung cancer
Source: Br J Cancer. 2020 Nov 16;124(4):713–20. doi: 10.1038/s41416-020-01055-5 (PMC7884704; doi:10.1038/s41416-020-01055-5)
Supplement: Supplementary file 1 — Supplementary File [file 41416_2020_1055_MOESM1_ESM.docx]

**Supplementary File**

Kang et al. A Randomised Phase IIb Study Comparing the Efficacy and Safety of Belotecan vs. Topotecan as Monotherapy for Sensitive-Relapsed Small-Cell Lung Cancer

| **Supplementary TABLE S1.** Baseline Characteristics of Patients for Efficacy Evaluation | | |
| --- | --- | --- |
|  | **Topotecan**  **(n = 76)** | **Belotecan**  **(n = 72)** |
| **Median age, years (range)** | 67 (42-83) | 64 (43-79) |
| **Median time to relapse, months (range)** | 7 (3-53) | 6 (3-51) |
| **Male, n (%)** | 62 (82%) | 58 (81%) |
| **Smoker, n (%)** | 66 (87%) | 62 (86%) |
| **Baseline Hb: < 12 g/dL, n (%)** | 45 (59%) | 49 (68%) |
| **ECOG PS: 1, n (%)** | 65 (86%) | 59 (82%) |
| **ECOG PS: 2, n (%)** | 1 (1%) | 2 (3%) |
| **ED at diagnosis, n (%)** | 46 (61%) | 43 (60%) |
| **ED at enrolment, n (%)** | 53 (70%) | 56 (78%) |
| **Metastatic disease, n (%)** | 66 (87%) | 62 (86%) |
| **Prior irinotecan use, n (%)** | 7 (9%) | 8 (11%) |
| **Prior surgery, n (%)** | 3 (4%) | 4 (6%) |
| **Prior radiotherapy, n (%)** | 53 (70%) | 53 (74%) |
| **Prior first-line treatment** |  |  |
| **Etoposide/Platinum, n (%)** | 70 (92%) | 63 (88%) |
| **Irinotecan/Platinum, n (%)** | 4 (5%) | 7 (10%) |

Abbreviations: ECOG PS, Eastern Cooperative Oncology Group performance status; Hb, haemoglobin; ED, extensive-stage disease.

**Supplementary TABLE S2.** Chemotherapy administered to subjects after the end of clinical drug administration

|  | **Belotecan**  **(n=80)** | | **Topotecan**  **(n=80)** | | **Total**  **(n=160)** | |
| --- | --- | --- | --- | --- | --- | --- |
|  | **n** | **%** | **n** | **%** | **n** | **%** |
| **Subject status at follow-up** | **80** |  | **80** |  | **80** |  |
| Unknown | 1 | 1.25 | 3 | 3.75 | 4 | 2.5 |
| Death | 70 | 87.5 | 70 | 87.5 | 140 | 87.5 |
| Survival | 9 | 11.25 | 7 | 8.75 | 16 | 10 |
| **Regimen change** | **80** |  | **80** |  | **80** |  |
| Yes | 47 | 58.75 | 35 | 43.75 | 82 | 51.25 |
| No | 25 | 31.25 | 33 | 41.25 | 58 | 36.25 |
| Unknown | 8 | 10 | 12 | 15 | 20 | 12.5 |
| **Modified Regimen** | **47** |  | **35** |  | **82** |  |
| Belotecan | 1 | 2.13 | 0 | 0 | 1 | 1.22 |
| Carboplatin | 0 | 0 | 2 | 5.71 | 2 | 2.44 |
| Cyclophosphamide+Doxorubicin+Vincristine | 6 | 12.77 | 7 | 20 | 13 | 15.85 |
| Etoposide | 1 | 2.13 | 0 | 0 | 1 | 1.22 |
| Etoposide+Carboplatin | 2 | 4.26 | 0 | 0 | 2 | 2.44 |
| Etoposide+Cisplatin | 4 | 8.51 | 1 | 2.86 | 5 | 6.1 |
| Gemcitabine+Cisplatin | 1 | 2.13 | 1 | 2.86 | 2 | 2.44 |
| Ifosfamide | 3 | 6.38 | 1 | 2.86 | 4 | 4.88 |
| Irinotecan | 0 | 0 | 3 | 8.57 | 3 | 3.66 |
| Irinotecan+Carboplatin | 7 | 14.89 | 6 | 17.14 | 13 | 15.85 |
| Irinotecan+Cisplatin | 13 | 27.66 | 9 | 25.71 | 22 | 26.83 |
| MTX | 1 | 2.13 | 0 | 0 | 1 | 1.22 |
| Nintedanib | 0 | 0 | 1 | 2.86 | 1 | 1.22 |
| Paclitaxel | 4 | 8.51 | 3 | 8.57 | 7 | 8.54 |
| Paclitaxel+Pembrolizumab | 1 | 2.13 | 0 | 0 | 1 | 1.22 |
| Pembrolizumab | 1 | 2.13 | 0 | 0 | 1 | 1.22 |
| Topotecan | 2 | 4.26 | 0 | 0 | 2 | 2.44 |
| Drugs for clinical trials | 0 | 0 | 1 | 2.86 | 1 | 1.22 |

**Supplementary TABLE S3**. Best overall response in the full analysis set (FAS), and in the intention-to-treat (ITT) and modified ITT (mITT) populations.

Best Overall Response (FAS, N=148)

|  |  | **Topotecan** | **Belotecan** | **Total** | **P-value^a^** |
| --- | --- | --- | --- | --- | --- |
|  |  | **(N=76)** | **(N=72)** | **(N=148)** |  |
| Objective Response Rate | N | 76 (100.00) | 72 (100.00) | 148 (100.00) | 0.0927 |
|  | CR or PR | 16 (21.05) | 24 (33.33) | 40 (27.03) |  |
|  | SD or PD | 60 (78.95) | 48 (66.67) | 108 (72.97) |  |
| Best Overall Response | N | 76 (100.00) | 72 (100.00) | 148 (100.00) | 0.0689 |
|  | CR | 0 (0.00) | 1 (1.39) | 1 (0.68) |  |
|  | PR | 16 (21.05) | 23 (31.94) | 39 (26.35) |  |
|  | SD | 37 (48.68) | 37 (51.39) | 74 (50.00) |  |
|  | PD | 23 (30.26) | 11 (15.28) | 34 (22.97) |  |
| a: Chi-square test or Fisher's exact test to compare ratios between groups | | | | | |

Best Overall Response (ITT, N=164)

|  |  | **Topotecan** | **Belotecan** | **Total** | **P-value^a^** |
| --- | --- | --- | --- | --- | --- |
|  |  | **(N=82)** | **(N=82)** | **(N=164)** |  |
| Objective Response Rate | N | 82 (100.00) | 82 (100.00) | 164 (100.00) | 0.1458 |
|  | CR or PR | 16 (19.51) | 24 (29.27) | 40 (24.39) |  |
|  | SD or PD or NA | 66 (80.49) | 58 (70.73) | 124 (75.61) |  |
| Best Overall Response | N | 82 (100.00) | 82 (100.00) | 164 (100.00) | 0.0910 |
|  | CR | 0 (0.00) | 1 (1.22) | 1 (0.61) |  |
|  | PR | 16 (19.51) | 23 (28.05) | 39 (23.78) |  |
|  | SD | 37 (45.12) | 37 (45.12) | 74 (45.12) |  |
|  | PD | 23 (28.05) | 11 (13.41) | 34 (20.73) |  |
|  | NA | 6 (7.32) | 10 (12.20) | 16 (9.76) |  |
| a: Chi-square test or Fisher's exact test to compare ratios between groups | | | | | |

Best Overall Response (mITT, N=161)

|  |  | **Topotecan** | **Belotecan** | **Total** | **P-value^a^** |
| --- | --- | --- | --- | --- | --- |
|  |  | **(N=81)** | **(N=80)** | **(N=161)** |  |
| Objective Response Rate | N | 81 (100.00) | 80 (100.00) | 161 (100.00) | 0.1325 |
|  | CR or PR | 16 (19.75) | 24 (30.00) | 40 (24.84) |  |
|  | SD or PD or NA | 65 (80.25) | 56 (70.00) | 121 (75.16) |  |
| Best Overall Response | N | 81 (100.00) | 80 (100.00) | 161 (100.00) | 0.1036 |
|  | CR | 0 (0.00) | 1 (1.25) | 1 (0.62) |  |
|  | PR | 16 (19.75) | 23 (28.75) | 39 (24.22) |  |
|  | SD | 37 (45.68) | 37 (46.25) | 74 (45.96) |  |
|  | PD | 23 (28.40) | 11 (13.75) | 34 (21.12) |  |
|  | NA | 5 (6.17) | 8 (10.00) | 13 (8.07) |  |
| a: Chi-square test or Fisher's exact test to compare ratios between groups | | | | | |

| Supplementary TABLE S4. Randomised Clinical Trials with Topotecan as Control for Second-line Treatment of Relapsed SCLC | | | | | |
| --- | --- | --- | --- | --- | --- |
| Clinicaltrials.gov identifier | **Trial phase** | **Experimental**  **Drug** | **Patient number** | **Trial**  **status** | **Note*^c^*** |
| NCT00319969 | 2 | Amrubicin | 76 (A)*^a^* | Completed |  |
| NCT00547651 | 3 | Amrubicin | 637 (A) | Completed | Benefit for refractory disease only |
| NCT00828139 | 2 | Ziv-aflibercept | 189 (A) | Completed | Similar efficacy, inferior safety |
| NCT01159327 | 2 | Sorafenib | 13 (A) | Terminated | Slow accrual |
| NCT01500720 | 2 | Cabazitaxel | 179 (A) | Completed | Inferior efficacy |
| NCT01533181 | 2 | Linsitinib | 44 (A) | Completed | Safe, lack of activity |
| NCT01803269 | 2 | Cyclodextrin-based polymer-camptothecin | 33 (A) | Terminated | Lack of activity, slow accrual |
| NCT01904253 | 2 | TAS-102 | 18 (A) | Terminated | Safety issue |
| NCT02200757 | 2 | Aldoxorubicin | 132 (E)*^b^* | Unknown |  |
| NCT02481830 | 3 | Nivolumab | 798 (A) | Active, not recruiting | Failed primary endpoint*^c^* |
| NCT02514447 | 2 | Trilaciclib | 120 (E) | Active, not recruiting |  |
| NCT02566993 | 3 | Lurbinectedin, Doxorubicin | 613 (A) | Active, not recruiting |  |
| NCT02738346 | 3 | Carboplatin, Etoposide | 164 (E) | Unknown |  |
| NCT02963090 | 2 | Pembrolizumab | 98 (E) | Active, not recruiting |  |
| NCT02980809 | 2 | Apatinib | 60 (E) | Not yet recruiting |  |
| NCT03059667 | 2 | Atezolizumab | 70 (E) | Active, not recruiting |  |
| NCT03061812 | 3 | Rovalpituzumab tesirine | 411 (E) | Recruiting |  |
| NCT03088813 | 3 | Irinotecan liposome injection | 486 (E) | Recruiting |  |
| NCT03098030 | 3 | Dinutuximab, Irinotecan | 470 (E) | Recruiting |  |
| NCT03554473 | 2 | M7824 | 53 (E) | Recruiting |  |

*^a^*(A), actual patient number.

*^b^*(E), estimated patient number.

*^c^*Information obtained from clinicaltrials.gov, publication, or press release.

**Supplementary FIGURE S1.** CONSORT patient flow diagram


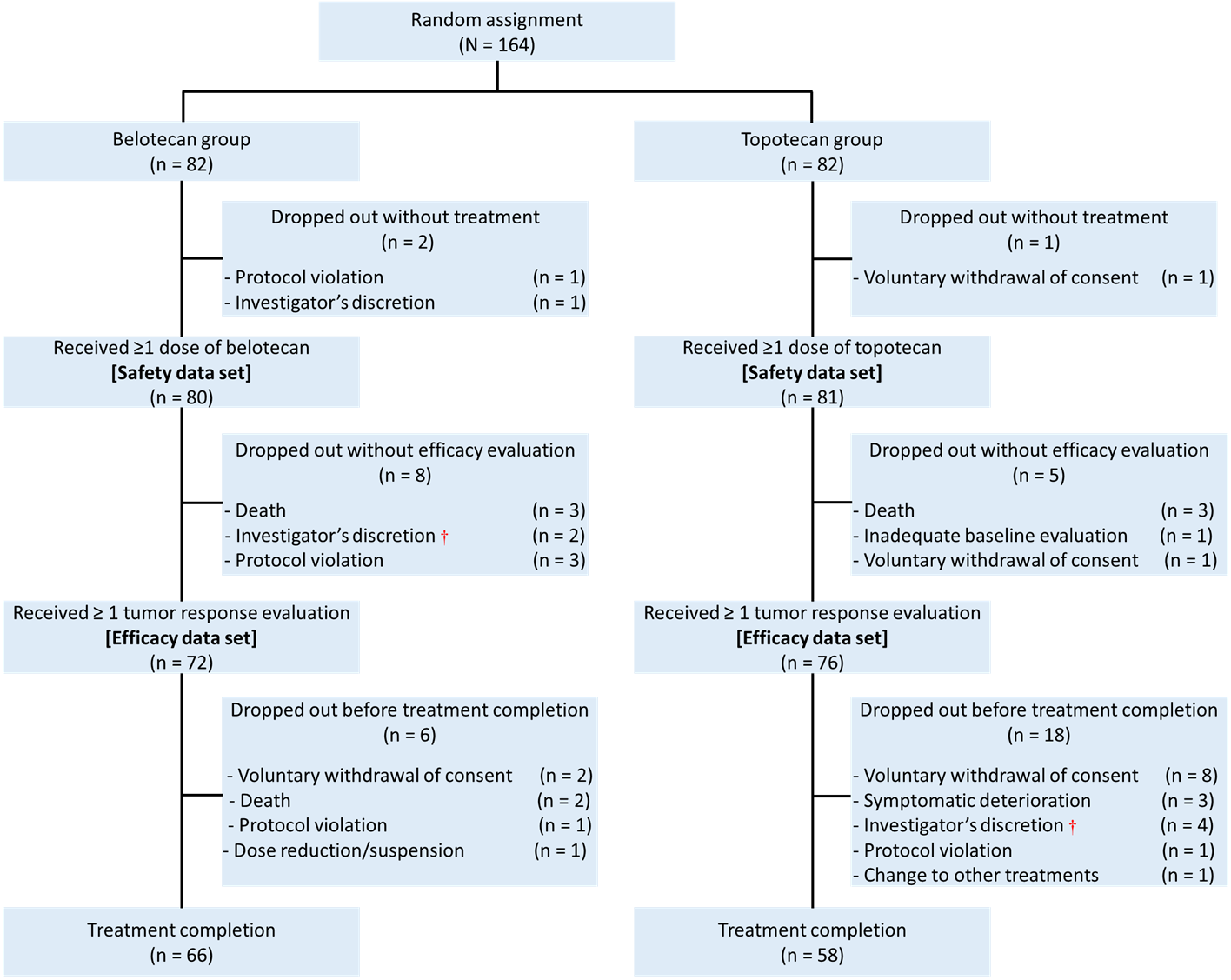


**Supplementary File.** Individual institutional review boards

| **Site Name** | **IRB No.** |
| --- | --- |
| National Cancer Center | NCCCTS-10-491 |
| The Catholic University of Korea, Seoul ST. Mary's Hospital | KC10MDMS0468 |
| Kangbuk Samsung Hospital | KBC10106 |
| Keimyung University Dongsan Medical Center | 10-83-2 |
| Seoul National University Hospital | H-1007-169-325 |
| Asan Medical Center | 2010-0479 |
| Severance Hospital | 4-2010-0366 |
| Ajou University School of Medicine | AJIRB-MED-CT2-10-191 |
| Chungbuk National University Hospital | 2010-07-045 |
| The Catholic University of Korea, ST. Vincent's Hospital | VC12MDMS0007 |
| KyungHee University Medical Hospital | KMC IRB 1312-02 |
| SMG-SNU Boramae Medical Center | 20130320/16-2013-48/041 |
| Chung-Ang University Hospital | C2013060(1020) |
